# Oncogenic Role of miR-200c-3p in High-Grade Serous Ovarian Cancer Progression via Targeting the 3′-Untranslated Region of DLC1

**DOI:** 10.3390/ijerph18115741

**Published:** 2021-05-27

**Authors:** Sheril June Ankasha, Mohamad Nasir Shafiee, Norhazlina Abdul Wahab, Raja Affendi Raja Ali, Norfilza Mohd Mokhtar

**Affiliations:** 1 Department of Physiology, Faculty of Medicine, Universiti Kebangsaan Malaysia, Jalan Yaacob Latif, Bandar Tun Razak, Cheras, Kuala Lumpur 56000, Malaysia; ankasha88@gmail.com (S.J.A.); hazlina@ukm.edu.my (N.A.W.); 2 Department of Obstetrics and Gynaecology, Faculty of Medicine, Universiti Kebangsaan Malaysia, Jalan Yaacob Latif, Bandar Tun Razak, Cheras, Kuala Lumpur 56000, Malaysia; nasirshafiee@hotmail.com; 3 Gastroenterology Unit, Department of Medicine, Faculty of Medicine, Universiti Kebangsaan Malaysia, Jalan Yaacob Latif, Bandar Tun Razak, Cheras, Kuala Lumpur 56000, Malaysia; draffendi@ppukm.ukm.edu.my

**Keywords:** microRNA, miR-200c, ovarian neoplasms, target site, luciferase assay, metastasis, epithelial-mesenchymal transition, 3′untranslated regions

## Abstract

High-grade serous ovarian cancer (HGSC) is the most common ovarian cancer with highly metastatic properties. A small non-coding RNA, microRNA (miRNA) was discovered to be a major regulator in many types of cancers through binding at the 3′-untranslated region (3′UTR), leading to degradation of the mRNA. In this study, we sought to investigate the underlying mechanisms involved in the dysregulation of miR-200c-3p in HGSC progression and metastasis. We identified the upregulation of miR-200c-3p expression in different stages of HGSC clinical samples and the downregulation of the tumor suppressor gene, *Deleted in Liver Cancer 1* (*DLC1*), expression. Over expression of miR-200c-3p in HGSC cell lines downregulated *DLC1* but upregulated the epithelial marker, *E-cadherin* (*CDH1*). Based on in silico analysis, two putative binding sites were found within the 3′UTR of *DLC1*, and we confirmed the direct binding of miR-200c-3p to the target binding motif at position 1488–1495 bp of 3′UTR of *DLC1* by luciferase reporter assay in a SKOV3 cell line co-transfected with vectors and miR-200c-3p mimic. These data showed that miR-200c-3p regulated the progression of HGSC by regulating *DLC1* expression post-transcription and can be considered as a promising target for therapeutic purposes.

## 1. Introduction

Ovarian cancer is the fifth most common cancer in women, and is the leading cause of death for patients with gynecological cancer. High levels of genetic and molecular heterogeneity are reported for epithelial ovarian cancer (EOC), and these epithelial cells can be transformed into different histological subtypes, either resembling the fallopian tube layer (serous, 52%), endometrium (endometrioid, 10%), endocervix glands (mucinous, 6%), or other vaginal cells (6%); the rest are rare histotypes [1,2].

Serous ovarian cancer (SOC) is classified into two categories, namely low-grade serous carcinoma (LGSC), which is a prototype for type I ovarian cancer and comprises less than five percent of EOC cases, and high-grade serous carcinoma (HGSC), which is a prototype for type II ovarian cancer. LGSC patients are often diagnosed at an early stage (stage I or II) with low mortality rates and tumor growth. Hence, the prognosis for this category is better, compared to HGSC [3,4,5]. About 70% to 80% of the total EOC subtypes are HGSC and are frequently associated with *TP53* mutation [6]. Patients are often diagnosed at a late stage, either stage III or IV, in which the tumor growth rate is aggressive and has spread to omentum and mesenteric areas. The condition leads to a low patient survival rate, with a 10-year mortality rate of 70% [2,4,5,7]. Therefore, extensive research and a better understanding of the molecular changes in the progression and metastatic process in HGSC are crucial, in order to develop a more effective therapeutic approach to overcoming this life-threatening disease [8,9].

MicroRNAs (miRNAs) are small non-coding RNA, approximately 20–22 nucleotides in length. They recognize and bind to partial complementary sequences of the messenger RNA (mRNA), commonly at the 3′ untranslated region (3′UTR) of the targeted gene, resulting in gene silencing through either mRNA cleavage or translation inhibition [10]. MiRNA plays an essential role in biological processes by negatively regulating gene expression of its targeted genes in normal and pathological cellular processes. In cancer, it can act either as oncogenic miRNAs (oncomiR) or tumor-suppressive miRNAs, based on their functional activities [11]. Growing evidence has indicated that the unique expression profiles for various cancer types could help in identifying tumor-specific miRNAs and their targeted genes for the development of novel molecular diagnostic and prognostic markers, especially in HGSC.

In order to predict miRNA targets, scientists frequently use in silico tools. This provides informative clues about miRNA biological targets, based on a number of criteria, such as complementary base-pairing, the thermodynamic stability of miRNA–mRNA duplex, conservation of target sites, and co-operative translation control and multiplicity of miRNA binding sites [12]. These in silico tools have enabled several predicted targets, with the assumptions that the sequences were functional sites [13].

Our previous miRNA profiling study identified a list of up and downregulated miRNAs in SOC and metastatic SOC (90% of samples were HGSC), where miR-200c-3p was significantly upregulated in SOC. *DLC1* is one of the target genes of miR-200c-3p, since miRNAs can have several distinct targets in the same context [14,15]. We also found that *Deleted in Liver Cancer 1* (*DLC1*) is the target gene, according to the in silico analysis [14]. In this current study, we decided to continue researching the role of miR-200c-3p regarding its targeted gene, *DLC1,* and tried to elucidate the underlying molecular mechanisms involved in the progression and metastasis of HGSC. An understanding of the interactions between miRNA–mRNA, and the validation of the targeted sites involved could potentially assist in miRNA-based drug development in the future.

## 2. Materials and Methods

### 2.1. Ethics Statement

The institutional ethics committee approved this study involving human tissue collection (reference no: UKM PPI/111/8/JEP-2017-268). All patients involved provided signed informed consent.

### 2.2. Human Tissues and Cell Lines

Twenty-nine specimens of early (stage I and II) and late (stage III and IV) stages of HGSC taken from primary ovarian tumor, and 21 unmatched normal ovarian surface tissues obtained from the Department of Obstetrics and Gynecology, Faculty of Medicine, Universiti Kebangsaan Malaysia Medical Centre (UKMMC), Malaysia, were snap frozen and stored at −80 °C. Written consent for tissue donation was obtained from every patient before surgery. The unmatched normal tissues were obtained from patients with a benign gynecological disorder who underwent total abdominal hysterectomy with bilateral salpingo-oophorectomy. Patients who underwent chemotherapy or those with secondary tumors were excluded from this study. All the cancer samples were then histologically verified by a pathologist using hematoxylin and eosin (H&E) staining to contain more than 80% malignant cells, while no cancer or inflammatory cells were detected in normal tissues.

Two human HGSC cell lines, primary tumor CAOV3 (ovarian adenocarcinoma) and metastatic SKOV3 (ovarian adenocarcinoma, ascites), were cultured in Dulbecco’s modified Eagle’s media in 4.5 g/L glucose (Invitrogen, Carlsbad, CA, USA) and McCoy’s 5A media (Invitrogen, Carlsbad, CA, USA) respectively, supplemented with 10% of fetal bovine serum (FBS) (Invitrogen, Carlsbad, CA, USA). Both cell lines were purchased from UKM Medical Molecular Biology Institute, Malaysia. All cell lines were maintained at 37 °C and 5% CO2 in a humidified incubator.

### 2.3. RNA isolation and quantitative Real Time-PCR (qRT-PCR)

Total RNA containing miRNA was isolated by using a miRNeasy kit (Qiagen, Germantown, CA, USA) according to the manufacturer’s protocol. The purity of the total RNA was assessed using a NanoDrop spectrophotometer (Thermo Scientific, Waltham, MA, USA) with criteria of purity met by A260/280 ratios of 1.8–2.1. Total RNA was then converted into cDNA and micDNA by using a RT2 First Strand kit and a miScript II RT kit (Qiagen, Germantown, CA, USA), respectively. Quantitative RT-PCR (qRT-PCR) was performed using an RT2 SYBR green PCR kit (Qiagen, Germantown, CA, USA) for mRNA and a miScript SYBR green PCR kit (Qiagen) for miRNA, using a CFX96 Real-Time System (BioRad, Hercules, CA, USA). We purchased primers for miR-200c-3p (catalog no. MS00003752) and the reference gene, RNU6 (catalog no. MS00033740), and primers for mRNA *DLC1* (catalog no. PPH00438B), *CDH1* (catalog no. PPH00135F), and the reference gene *PPIA* (catalog no. PPH01319G) from Qiagen. The relative expression was calculated using the 2-ΔΔCT method.

### 2.4. Transient miR-200c-3p transfection

Each HGSC cell line, CAOV3 and SKOV3, was cultured in 6-well plates with 5 × 105 cells/well with antibiotic-free culture medium until reaching 70% confluence. Cells were then transiently transfected with 150 nM (with transfection efficiency 90%, Supplementary Figure S1) miRCURY LNA™ miR-200c-3p mimic or inhibitors, and their respective negative control (Exiqon, Vedbaek, Denmark), together with Lipofectamine^®^ RNAimax (Invitrogen) and Opti-MEM^®^ I without serum and antibiotic (Invitrogen, Carlsbad, CA, USA) for 48 h. Cells were then collected and we immediately continued with RNA extraction and protein extraction. The *DLC1* mRNA and protein expression analyses were made using qRT-PCR and Western blot, respectively. All treatments were tested in biological triplicate.

### 2.5. Protein Extraction and Western Blot Assay

Each cell line was lysed with RIPA buffer with 0.1 M dithiothreitol, protease, and phosphatase inhibitors (Thermo Scientific, Waltham, MA, USA). The total protein was quantified using a BCA protein assay (Thermo Scientific, Waltham, MA, USA). The protein (25 µg) from each sample was loaded into 10% SDS-PAGE gel, separated by electrophoresis, and transferred onto a PVDF membrane (Biorad, Hercules, CA, USA). The membrane was blocked with 5% skimmed milk and incubated with primary antibody against DLC1 (Santa Cruz, CA, USA) and β-actin (Abcam, Cambridge, MA, USA) at 1:2000 overnight at 4 °C. Incubation with secondary antibody HRP (Abcam, Cambridge, MA, USA) at 1:5000 was performed on the next day for an hour at room temperature and visualized by chemiluminescence (ECL, Abcam, USA) with a gel doc Amersham Imager 6000 (GE Life Sciences, Buckinghamshire, UK). Band intensity on the PVDF membranes was analyzed with Image J software and normalized to β-actin level.

### 2.6. Prediction of miRNA target sites in the 3′UTR of DLC1

There are many methods and web-based services recently developed for the integrated analysis of miRNA and mRNA expression data. MicroRNA.org [16] and TargetScan [17] were used to find the target prediction of miR-200c-3p on the 3′UTR of *DLC1*.

### 2.7. Cloning of the 3′UTR of DLC1

The 3′UTR of *DLC1* (nucleotides 4842–7412 of NM_182643.3) containing two putative miR-200c-3p binding sites were amplified from human genomic DNA. Then, reverse complement of 3′UTR of *DLC1*, and two additional wild-type (WT) sequences containing one of the binding sites were synthesized by PCR with the primers (Table 1). All forward and reverse primers contained SbfI and XbaI restriction enzyme sequence (underlined), respectively, at the 5′ end to produce flanking restriction sites at the end of the PCR products to facilitate cloning into the pmirGLO dual-luciferase miRNA target expression vector (Promega, Madison, WI, USA). All the amplicons and vectors were digested with SbfI and XbaI, ligated, and transformed into 10-beta competent *E. coli* (New England Biolabs, Ipswich, MA, USA) for cloning purposes.

Three additional sequences of 3′UTR with a mutant type (MT) deletion for both binding sites (MT-AB) and each of the two binding sites (MT-A and MT-B) were synthesized by PCR using a Phusion Site-Directed Mutagenesis Kit (Thermo Fisher Scientific, USA) with the following pair of primers: MT-A forward primer 5′-TTGTGTATGGATCAAAAGTG-3′ and reverse primer ‘5′-GAGTCAATCCTTACCATTAC-3′, MT-B forward primer 5′-TTGTACTTGTAGGCACCTAATAAATTTATTATTTGC-3′ and reverse primer 5′-AAAGCCGCTGCAGGGGAT-3′, and for MT-AB, primers’ MT-A were used with the template from MT-B.

Another four constructs with an isolated sequence containing two small regions of 3′UTR, namely, WT_A (46bp) and WT_B (53bp), and two mutant-type constructs with substitution mutated on the first three nucleotides of the seed sequence, i.e., MT_A (46bp) and MT_B (53 bp) were constructed (Table 2). All four vectors were constructed and validated by Gen Script, USA.

### 2.8. Luciferase Reporter Assay

SKOV3 cells (4 × 105 cells/well) were cultured in 96-well plate and co-transfected with three pmol miR-200c-3p mimic or negative control, and 100 ng of either wild-type or mutant-type pmirGLO plasmid for 48 h using Lipofectamine 2000 (Invitrogen, Carlsbad, CA, USA). Cells were then assayed with a Dual-Luciferase Reporter Assay System (Promega, Madison, WI, USA) according to the manufacturers protocols. All treatments were tested in biological triplicate.

### 2.9. Statistical Analysis

All data were expressed as mean ± standard error of mean (SEM). Statistical analyses were performed using GraphPad Prism 5.0 (GraphPad Software, Inc., La Jolla, CA, USA). Student’s t-test analyzed the statistical significance between groups. Pearson’s correlation analysis determined the correlation between miR-200c-3p vs. *DLC1*.A difference with *p* < 0.05 was considered to be statistically significant.

## 3. Results

### 3.1. Clinical and Pathological Characteristics of Samples

We collected 29 primary ovarian tumor tissues from HGSC patients (17 and 12 samples were at the early and late stage, respectively) who met the inclusion criteria and 21 unmatched normal ovarian surface epithelium tissues from patients with benign gynecological diseases who underwent total abdominal hysterectomy with bilateral salpingo-oophorectomy, as the control. All the tissues obtained were histologically validated using hematoxylin and eosin (H&E) staining by a pathologist to confirm that the normal ovary tissue samples were free from cancer or inflammatory cells and that the cancer tissues contained more than 80% malignant cells. The inclusion criteria included patients with primary epithelial ovarian cancer, and who had not undergone chemotherapy. Table 3 shows a summary of the collected clinical samples.

### 3.2. Expression Levels of miR-200c-3p and DLC1 in Clinical Specimens.

**Figure 1:**
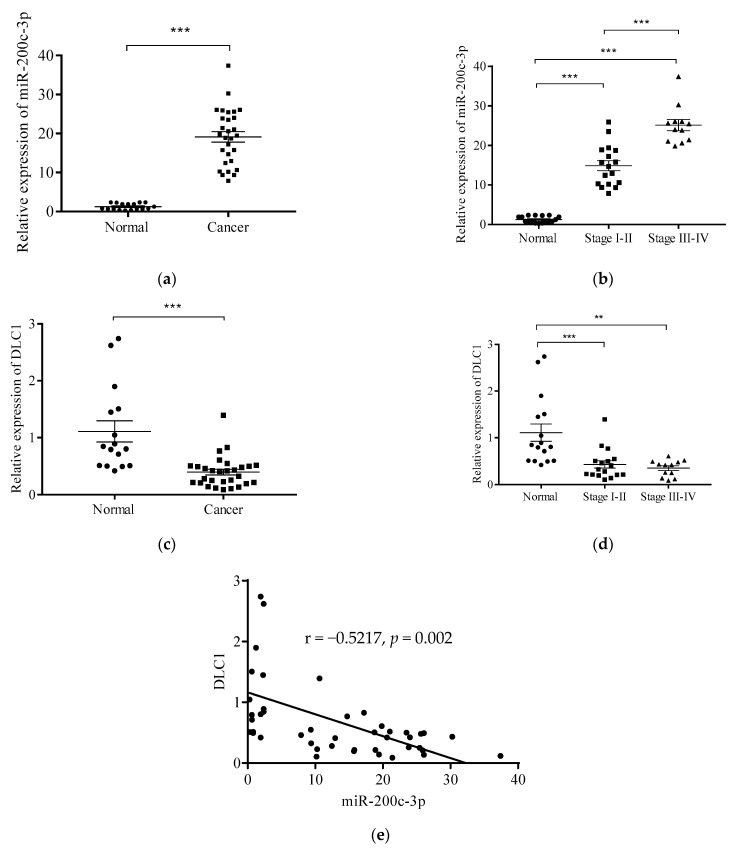
The expression levels of miR-200c-3p and *DLC1* are inversely correlated in high grade serous carcinoma. The miR-200c-3p expression by qRT-PCR showing upregulation in cancer tissues (**a**), and different stages of HGSC (**b**). Conversely, the expression level of *DLC1*, showing downregulation in cancer tissues (**c**), and different stages (**d**). A negative correlation was shown between miR-200c-3p and *DLC1* in both early and late stage of high grade serous carcinoma samples (**e**). Significant values are indicated as follows: ** *p* < 0.01 and *** *p* < 0.001.

The relative expression level of miR-200c-3p in HGSC tissues was found to be significantly upregulated, by 19.13-fold compared to normal tissues (*p* < 0.0001) (Figure 1a). The trend of the relative expression level of this miRNA was found to be significantly upregulated in early stage (I and II) by 14.89-fold, and followed by the late stages (III and IV) by 25.14-fold as compared to the normal (*p* < 0.0001). This showed the expression of miR-200c-3p increased with the progression of the disease (Figure 1b).

On the other hand, the relative expression level of *DLC1* in cancer tissues was found to be significantly downregulated, by 0.4-fold compared to the normal tissues (*p* < 0.0001) (Figure 1c). Similar results were found in different stages of HGSC, where the relative expression level was downregulated significantly in both early, by 0.37-fold (*p* < 0.001), and late stage, by 0.35-fold (*p* < 0.01), compared to normal (Figure 1d).

The Pearson correlation coefficient (r) between the relative expression levels of miR-200c-3p and *DLC1* was found to have a moderate correlation, with the value of r = −0.5217 and *p* = 0.002 (Figure 1e). This result showed that there was a negative correlation between miR-200c-3p and *DLC1* expression, which suggested *DLC1* was a target of miR-200c-3p in the HGSC samples.

### 3.3. DLC1 Is a Direct Target of miR-200c-3p in an In Vitro Model of HGSC

**Figure 2:**
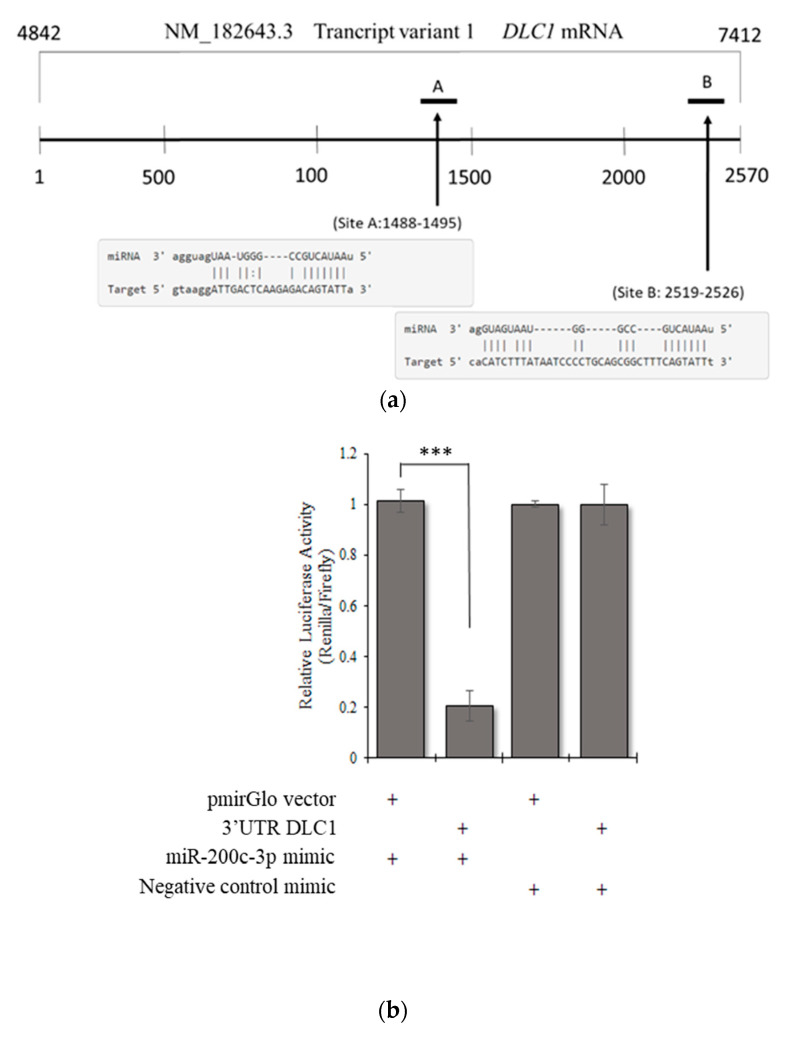
(**a**) Two putative binding sites of 3′UTR *DLC1* from in silico analysis. (**b**) There was a significant reduction in the luciferase activity when 3′UTR DLC1 construct was co-transfected with miR-200c-3p mimic. Significant values are indicated as follows: *** *p* < 0.001.

The bioinformatic prediction tools, microRNA.org (http://www.microrna.org, accessed on 20 May 2021) and TargetScan (http://www.targetscan.org, accessed on 20 May 2021) were accessed on 6 February 2019 have suggested that there are two putative binding sites for miR-200c-3p in the 3′UTR of *DLC1* (Supplementary Figure S2), which are an 8-mer seed CAGTATTA (1488–1495 bp from the start of 3′UTR) and a 7-mer-m8 seed CAGTATT (2519–2526 bp from the start of 3′ UTR) of the transcript variant 1, as shown in Figure 2a. To investigate the relationship between miR-200c-3p and *DLC1* expression through miRNA:mRNA binding at the 3′UTR, a firefly luciferase reporter vector was constructed by inserting a wild-type (WT) 3′UTR of *DLC1* containing two binding sites in the 3′ end of the luciferase gene and the sequence was confirmed by Sanger sequencing (Supplementary Figure S3). The data indicated that overexpression of miR-200c-3p remarkably reduced the luciferase activity of DLC1 3′UTR by 80% (*p* < 0.0001) (Figure 2b).

**Figure 3:**
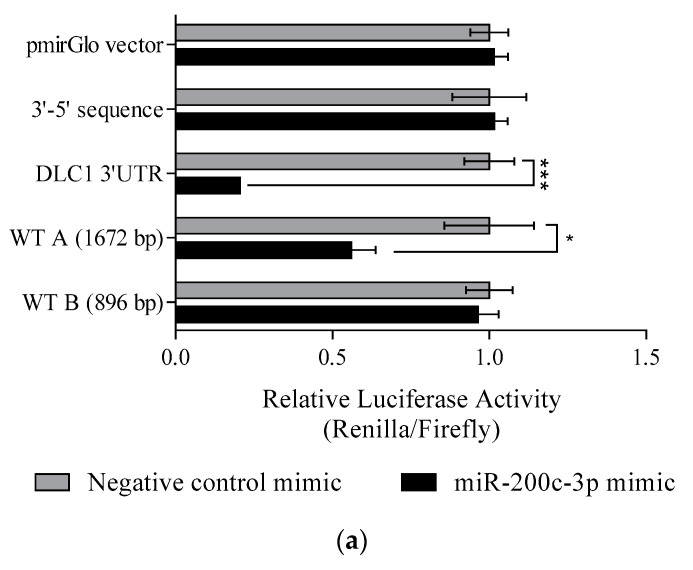

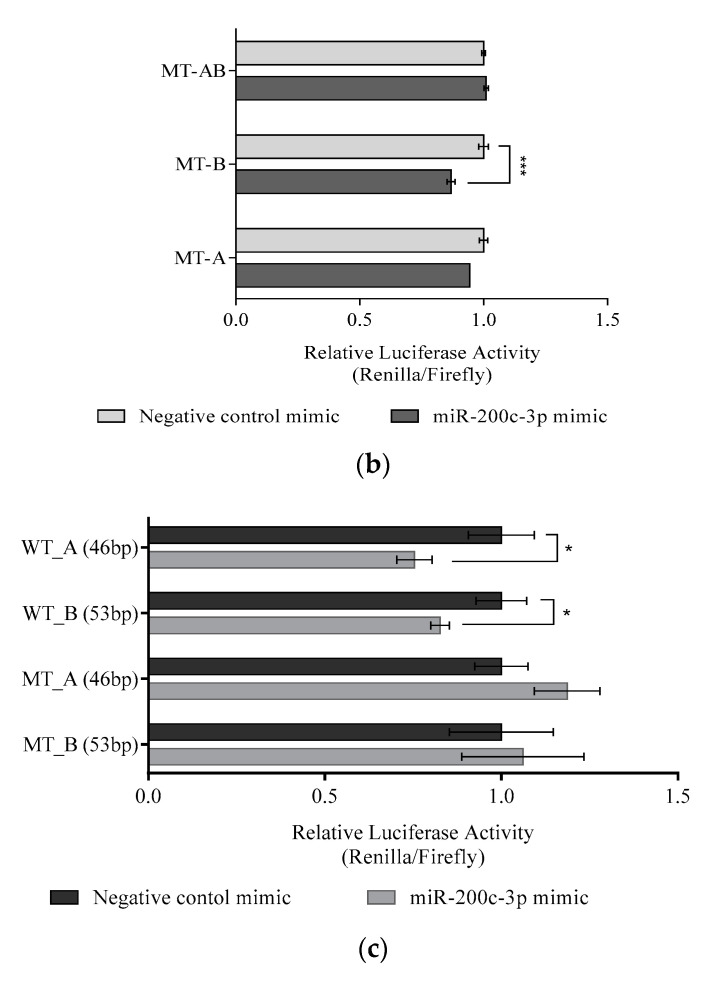
(**a**) Luciferase activity showed a significant reduction in vector containing DLC1 3′UTR (*p* < 0.0001) and WT-A (*p* < 0.05) but not in WT-B. (**b**) MT-B showed a significant reduction (*p* < 0.001) in luciferase activity, whereas there was no significant reduction in MT-A, and in MT-AB with the presence of miR-200c-3p. (**c**) With the presence of miR-200c-3p, luciferase activity showed a significant reduction in both wild-type vectors, WT-A (46bp) and WT-B (53bp), but not in mutant-types. Significant values are indicated as follows: * *p* < 0.05 and *** *p* < 0.001.

However, in order to identify the specific binding area of miR-200c-3p on DLC1 3′UTR, two firefly luciferase reporter vectors containing site A or site B, namely WT-A or WT-B, respectively, were constructed. Figure 3a shows that overexpression of miR-200c-3p significantly reduced the luciferase activity, by 44%, in WT-A (*p* < 0.05), but not in WT-B. This showed that the binding area of miR-200c-3p could be within the 1672 bp of WT-A.

We further investigated whether these two binding sites were repressed by miR-200c-3p; three additional luciferase vectors were constructed containing the mutant DLC1 3′UTR sequence with deletions for both binding sites (MT-AB) and either of the two binding sites (MT-A or MT-B). As shown in Figure 3b, MT-A and MT-AB attenuated the inhibitory effects on the luciferase activity in the presence of miR-200c-3p mimic. However, the luciferase activity was significantly inhibited for MT-B construct of the luciferase activity in the presence of miR-200c-3p mimic (*p* < 0.001). To be more specific, the inhibition of luciferase activity was mainly observed by the first putative binding site, which is site A, since the deletion could result in the loss of over 90% of luciferase inhibitory activity. These results confirmed that miR-200c-3p repressed the *DLC1* gene through its binding in the 3′UTR at site A.

To confirm the specific target binding site of miR-200c-3p on 3′UTR, a small region containing 46 bp for site A, and 53 bp for B was isolated into different vectors, namely, WT-A (46bp) and WT-B (53bp), respectively. Additionally, two vectors containing three substitution mutations on the first three nucleotides (5′–3′) of seed sequences were constructed. With the presence of miR-200c-3p, luciferase activities were significantly reduced by 25% and 20% in both WT-A (46bp) and WT-B (53bp), respectively (*p* < 0.05). However, the inhibitory effects were attenuated in both mutant-type containing vectors (Figure 3f). All the constructed vectors are shown in Supplementary Figure S4.

### 3.4. Regulation of DLC1 by miR-200c-3p in HGSC Cell Lines

**Figure 4:**
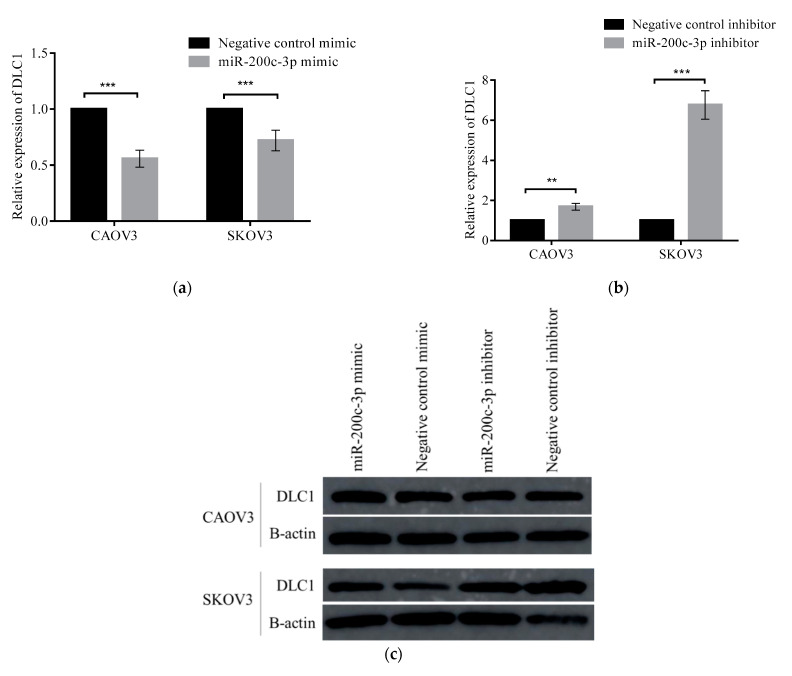
The expression of DLC1 in CAOV3 and SKOV3 cells. (**a**) After 48 h of miR-200c-3p mimic transfection, *DLC1* mRNA levels were significantly reduced compared to negative control mimic. (**b**) Conversely, *DLC1* mRNA expression levels increased with miR-200c-3p inhibitor, compared to negative controls. (**c**) Western blot assay showed no significant difference in DLC1 protein expression in both CAOV3 and SKOV3 cells. Significant values are indicated as follows: ** *p* < 0.01 and *** *p* < 0.001.

To obtain additional evidence on the role of miR-200c-3p towards *DLC1*, we investigated further, by transiently transfected miR-200c-3p mimic or inhibitor in HGSC cell lines, which were CAOV3 (primary tumor) and SKOV3 (isolated from ascites), together with their respective negative controls. Figure 4a shows that the relative expression of *DLC1* mRNA was significantly downregulated (*p* < 0.001) in both CAOV3 and SKOV3 cell lines transfected with miR-200c-3p mimic, by 0.06-fold and 0.72-fold, respectively. On the other hand, the relative expression of *DLC1* mRNA was shown to be significantly upregulated in both CAOV3 cells, by 1.69-fold (*p* < 0.01), and SKOV3 cells, by 6.76-fold (*p* < 0.0001), after transfection with miR-200c-3p inhibitor (Figure 4b). However, there was no significant difference in DLC1 protein level in both cell lines transfected with miR-200c-3p mimic or inhibitor compared to the control (Figure 4c). These results gave a reliable indicator of the role of miR-200c-3p in regulating *DLC1* at the mRNA level in HGSC progression.

### 3.5. Regulation of miR-200c-3p in Epithelial-Mesenchymal Transition through Modulation of DLC1 Expression

**Figure 5:**
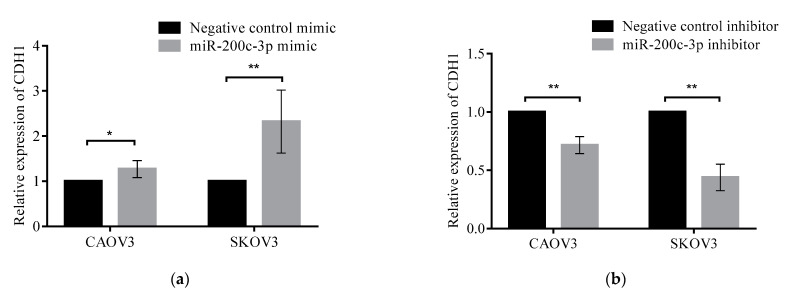
The expression level of EMT/MET markers detected by quantitative real-time qRT-PCR in HGSC cell lines. (**a**) The CAOV3 and SKOV3 cells transfected with miR-200c-3p mimic showed a significantly increased expression level of *CDH1*. (**b**) Following transfection with miR-200c-3p inhibitor, there was significant downregulation of *CDH1* expression. Significant values are indicated as follows: * *p* < 0.05 and ** *p* < 0.01.

To explore the association between miR-200c-3p regulation on *DLC1* and its effect on the progression and metastasis of HGSC, CAOV3 and SKOV3 cell lines were transiently transfected with miR-200c-3p mimic or inhibitor. The relative expression levels of EMT/MET markers, *E-cadherin* (*CDH1*), in addition to *DLC1* were assessed in comparison to their respective negative controls. Figure 5a shows that in the presence of miR-200c-3p mimic, the relative expression of *CDH1* was significantly upregulated in CAOV3 cells, by 1.27-fold (*p* < 0.05), and in SKOV3 cells, by 2.32-fold (*p* < 0.01), in comparison to the controls. However, in Figure 5b, when the cells were transfected with miR-200c-3p inhibitors, there was a significant downregulation in the expression of *CDH1* in both CAOV3 and SKOV3 cells, by 0.72-fold and 0.44-fold, respectively (*p* < 0.01). These results showed that miR-200c-3p could be a regulator for EMT/MET through the modulation of *DLC1* expression, which plays an essential role in HGSC progression and metastasis.

## 4. Discussion

Increasing evidence has shown that aberrant changes in miRNAs, especially the miR-200 family, play critical roles in the progression and carcinogenesis of ovarian cancer [18,19]. Previously, we shortlisted upregulated miRNAs in SOC samples using miRNA microarray analysis, where miR-200c-3p was the most upregulated in SOC compared to normal ovarian tissues. Even though both low-grade and high-grade SOC were used in the study, more than 90% of the samples were high-grade [14]. Thus, in the present study, we further evaluated the expression of miR-200c-3p in different stages of HGSC compared to normal tissues, and identified the binding region at 3′UTR of the targeted gene.

In this study, we clearly proved that the expression pattern of miR-200c-3p in HGSC tissues at different stages was found to parallel the aggressiveness of the cancer cells, where the expression was the highest in the late stages. These results were in concordance with a previous study that showed miR-200c-3p was significantly associated with the advanced stages of HGSC [18], lymph node metastasis, and poor clinical outcome [20]. Simultaneously, there was a downregulation of the tumor suppressor gene, *DLC1,* a computational prediction gene identified from the bioinformatic analysis.

*DLC1* (also known as ARHGAP7 and STARD12 in humans) is a gene that encodes the RhoGAP protein critically involved in cytoskeleton regulation, angiogenesis, and cell migration by inactivating Rho GTPase [21,22]. It is a tumor suppressor gene that could be dysregulated by genetic and epigenetic mechanisms in many types of cancers, such as lung, breast, gastric, liver, and ovarian cancer [23,24,25,26,27]. From a meta-analysis study, it was shown that *DLC1* expression was downregulated in the advanced stages compared to the early-stage cancer in 19 eligible studies, including epithelial ovarian cancer (EOC), but those studies did not mention the histological subtypes of EOC [28]. Hence, our findings showed that *DLC1* was the most downregulated in the late stage of HGSC and might be associated with high expression of miR-200c-3p through its direct binding at 3′UTR. Furthermore, our findings confirmed that there was a negative correlation between miR-200c-3p and *DLC1* expression in HGSC tissues.

From the bioinformatic analysis, it was found that there are two putative miR-200c-3p binding sites within the 3′UTR of *DLC1*. For the first time, in the present study, we verified and confirmed miR-200c-3p direct binding on 3′UTR of *DLC1* in luciferase reporter experiments in SKOV3 cell line. Based on these results, the main binding site was found to be at the first site of 3′UTR, a conserved target site with an 8-mer seed sequence, 5′-CAGTATTA-3′. This was then subsequently verified by mutating the target site, which retracted the binding actions by miR-200c-3p. This study was in an agreement with a previous study, where in an UTR fragment containing two sites, the repression at the 8-mer site was the most favorable. However, the repression at both sites in one UTR fragment was greater than the expected site individually [29]. This could be due to synergistic effects, for which the exact mechanism remains unknown [29,30].

To overcome the ‘off-target’ effects which occur through the same identification based on seed recognition as endogenous miRNA targeting, gain and loss of miRNA function tests are required [29]. Herein, we investigated the expression of miR-200c-3p in HGSC cell lines, and the results confirmed that miR-200c-3p downregulated *DLC1,* and that when miR-200c-3p expression was inhibited, *DLC1* expression increased. However, this in vitro study demonstrated that the downregulation of *DLC1* in the presence of over expression of miR-200c-3p only took place at an mRNA level. Hence, the regulation of miR-200c-3p at 3′UTR of *DLC1* would cause mRNA degradation instead of translation repression. Nonetheless, this is still unclear, due to the complexity of the underlying mechanisms involved.

EMT and MET, an epithelial to mesenchymal transition and vice versa, are the transdifferentiations between epithelial cells and motile mesenchymal cells, which is a critical process in metastasis. Multiple established markers served as core markers in the assessment of EMT/MET, such as up- or downregulation of *CDH1*, a hallmark of EMT that disrupts adherens junctions, and which is customarily accompanied by a reduction in the expression of *CDH2*, *VIM,* and others [31,32]. A previous study demonstrated that over expression of *DLC1* could inhibit EMT by increasing the expression of epithelial markers [33]. According to another report, the inhibitory effects of *CDH1* could be attenuated by knocking down *DLC1* epigenetically [34]. However, the present study displayed contradictory results, which showed a negative regulation between *DLC1* and *CDH1* when miR-200c-3p was overexpressed.

In this study, even though the high expression of miR-200c-3p was found to cause downregulation of a tumor suppressor gene *DLC1*, at the same time it also led to upregulation of *CDH1* expression in both primary and metastatic tumor cells. Based on this data, we suggested that over expression of miR-200c-3p could reduce *DLC1* mRNA and hence, inhibited EMT and promoted MET in HGSC, as observed by the upregulation of the epithelial marker, E-cadherin. Our previous study confirmed that over expression of miR-200c stimulated cell proliferation and colony formation but attenuated cell migration and invasion [14]. Moreover, MET could also be regulated by miR-200c through a double feedback loop with the two E-cadherin transcriptional regulators, ZEB1 and ZEB2, and influencing TGF-β and EGFR signaling cascades [35,36]. Eventually, this caused cells to re-epithelialize to form metastatic foci at a secondary site.

The use of the SKOV3 cell line might be a possible limitation of our study. The SKOV3 cells was mostly referred as ‘serous’ due to some uncertainties about putative *TP53* mutation. However, the genetic analysis showed that SKOV3 not only had a mutation on *TP53*, but also in *ARID1A,* where it was most likely to be endometrioid or clear cell carcinoma [37,38]. Therefore, a better-defined system model with valid clinical phenotypes needs to be considered in future studies.

In the future, miR-200c-3p could have a great potential as a diagnostic marker for early detection, as well as a prognostic marker to complement the current cancer staging system in ovarian cancer [39,40]. Furthermore, for therapeutic purposes, with the target sequence known, it could be proposed to form an miRNA sponge containing binding sites of the target gene to compete with the ‘authentic target’ [41]. The use of miRNA as a target in medical therapies demonstrates its role as an alternative in combating debilitating cancers, such as ovarian cancer

## 5. Conclusions

In conclusion, upregulated miR-200c-3p expression in both early and late stage of HGSC could downregulate *DLC1*, as a tumor suppressor. At the same time, miR-200c-3p could regulate EMT/MET in HGSC cells. Therefore, we suggest that a combination of miR-200c-3p and the molecular target sites of *DLC1* could be a potential regulator in HGSC for therapeutic purposes and to reverse cancer progression in this deadly disease.

## Figures and Tables

**Table 1:** The primer sequences for DLC1 3′UTR, WT-A, WT-B, and 3′-5′ inserts. The underlined sequence is the restriction enzyme sequence.

Insert	Forward Primer with XbaI (5′ to 3′)	Reverse Primer with SbfI (5′ to 3′)	Size of Amplicon
DLC1 3′UTR	TAAGCATCTAGAGGCCACATGCCAGAATG	TGCTTACCTGCAGGCAGTATAGCAAATAATAAATTTATTAGGTG	2582 bp
WT_A	TAAGCATCTAGAGGCCACATGCCAGAATG	TGCTTACCTGCAGGCACTGATATCCAAAATACTCAAATTTTAA	1684 bp
WT_B	TAAGCATCTAGATTCCTCATGAAGATATACATGGA	TGCTTACCTGCAGGCAGTATAGCAAATAATAAATTTATTAGGTG	900 bp
3′–5′	TAAGCATCTAGACAGTATAGCAAATAATAAATTTATTAGGTG	TGCTTACCTGCAGGGGCCACATGCCAGAATG	2582 bp

**Table 2:** The 3′UTR of *DLC1* target site isolation and substitutional mutations. The underlined sequence is the miR-200c-3p seed sequence, and the red sequence is the substitution mutation sequence.

Construct	Sequence	Size
WT_A (46bp)	5′-AAGAGACAGTATTAGTAAA-3′	46 bp
MT_A (46bp)	5′-ACTCAAGAGAGTCTATTAGTAA-3′	46 bp
WT_B (53bp)	5′-GCGGCTTTCAGTATTTTGTACT-3′	53 bp
MT_B (53bp)	5′-CGGCTTTGTCTATTTTGTAC-3′	53 bp

**Table 3:** Summary of clinical samples.

Characteristics	Cancer Samples (*n* = 29)	Normal Samples (*n* = 21)
Age (year)		
Age (median)	54 (24,80)	55 (35,80)
≤54	14 (48.23%)	10 (47.62%)
>54	15 (51.72%)	11 (52.38%)
Race		
Malay	18 (62.07%)	11 (52.38%)
Chinese	10 (34.48%)	9 (42.86%)
Others	1 (3.45%)	1 (4.76%)
Stage		
I	11 (37.93%)	
II	6 (20.69%)	
III	8 (27.59%)	
IV	4 (13.79%)	

## Supplementary Materials

The following are available online at https://www.mdpi.com/article/10.3390/ijerph18115741/s1, Supplementary Figure S1. Transfection efficiency of miR-200c-3p mimic in CAOV3 (A) and SKOV3 cells (B). Both cells showing more than 90% efficiency in 150 nM. Supplementary Figure S2. Predicted miR-200c-3p binding regions located in human 3′UTR DLC1 derived from TargetScan Release 7.1 (A) and microrna.org (B). Supplementary Figure S3. (A) Cloning of 2.57 kb of DLC1 3′UTR with seed sequences highlighted in green at XbaI-SbfI sites (highlighted in red) in the pmirGlo reporter vector. (B) Sequencing analysis confirmed seed sequences cloned in the vector. Supplementary Figure S4. (A) Representation of vectors with each of the binding sites constructed, namely WT-A and WT-B. (B) The constructs for luciferase activity of vectors containing, DLC1 3′UTR sequence, deletions of both site A (MT-A) and B (MT-B) or either site A or B (MT-AB). (C) Isolation of the binding site sequence (underlined) constructed into different vectors namely WT-A (46 bp) and WT-B (53 bp) together with mutant type vectors contained three substitutional mutation (red sequence) namely MT-A (46 bp) and MT-B (53 bp).
