# Supplementary material for: Oncogenic Role of miR-200c-3p in High-Grade Serous Ovarian Cancer Progression via Targeting the 3′-Untranslated Region of DLC1
Source: Int J Environ Res Public Health. 2021 May 27;18(11):5741. doi: 10.3390/ijerph18115741 (PMC8198916; doi:10.3390/ijerph18115741)
Supplement: Supplementary file 1 [file ijerph-18-05741-s001.zip › ijerph-1187033-supplementary.pdf]

Supplementary Figure 1. Transfection efficiency of miR-200c-3p mimic in (A) CAOV3 and (B) SKOV3 cells. Both cells showing more than 90% efficiency in 150 nM

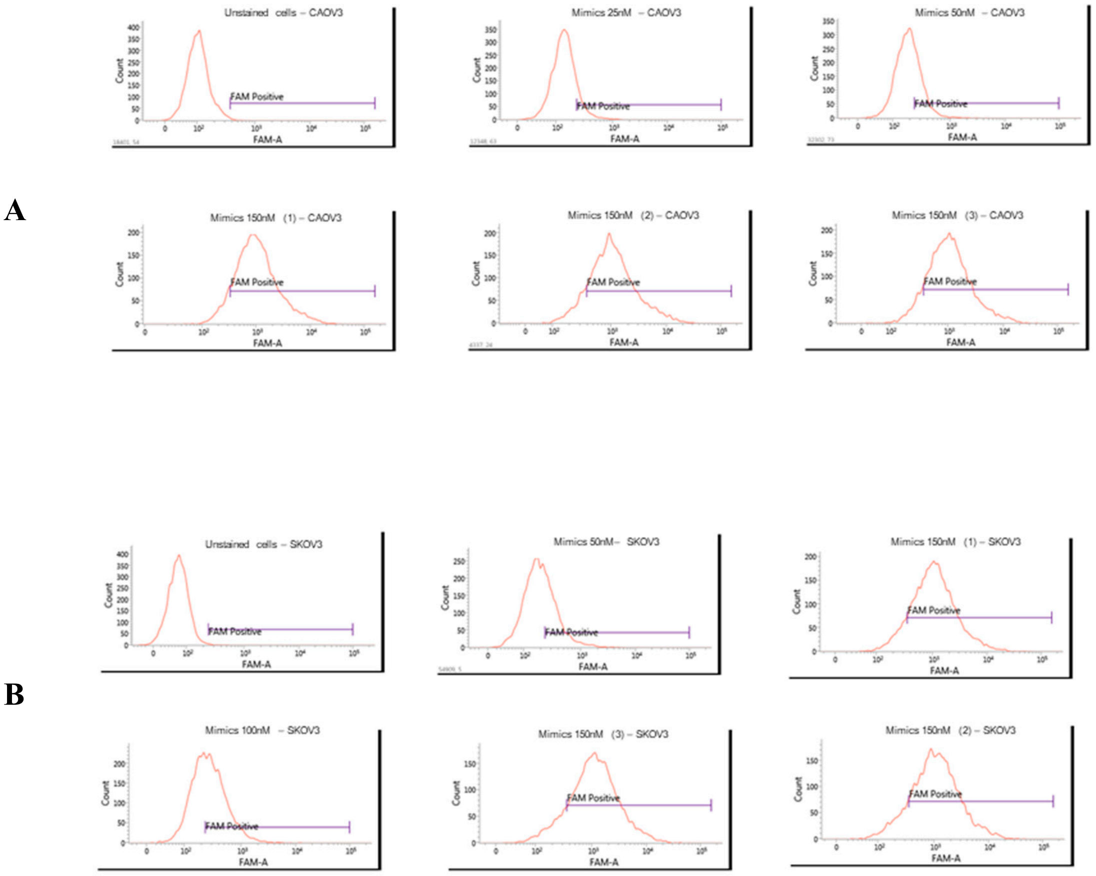

Supplementary Figure 2. Predicted miR-200c-3p binding regions located in human 3'-UTR *DLC1* derived from (A) TargetScan Release 7.1 and (B) microrna.org

A

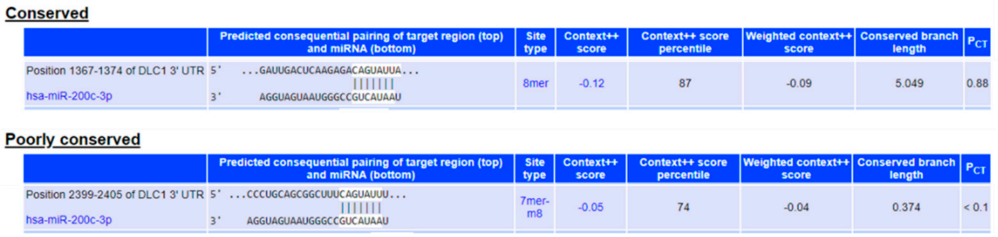

Conserved

	Predicted consequential pairing of target region (top) and miRNA (bottom)	Site type	Context++ score	Context++ score percentile	Weighted context++ score	Conserved branch length	P_{CT}
Position 1367-1374 of DLC1 3' UTR<br>hsa-miR-200c-3p	5' ...GAUUGACUCAAGAGACAGUAUUA...<br>||||||| 3' AGGUAGUAAUGGGCCGUCAUAAU	8mer	-0.12	87	-0.09	5.049	0.88

Poorly conserved

	Predicted consequential pairing of target region (top) and miRNA (bottom)	Site type	Context++ score	Context++ score percentile	Weighted context++ score	Conserved branch length	P_{CT}
Position 2399-2405 of DLC1 3' UTR<br>hsa-miR-200c-3p	5' ...CCCUGCAGCGGCUUUCAGUAUUU...<br>||||||| 3' AGGUAGUAAUGGGCCGUCAUAAU	7mer-m8	-0.05	74	-0.04	0.374	< 0.1

B

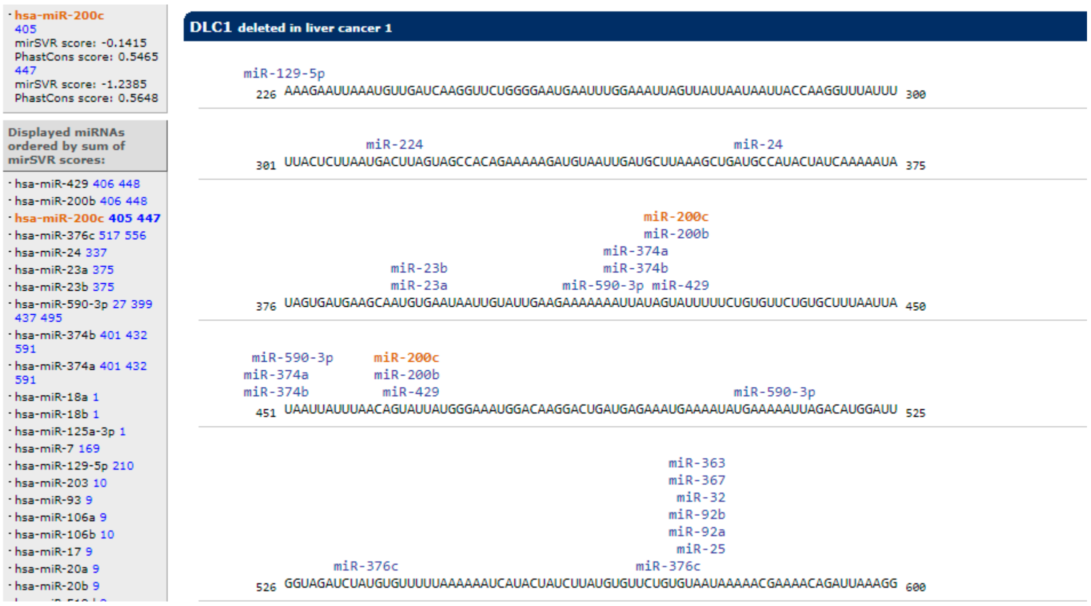

Supplementary Figure 3. (A) Cloning of 2.57 kb of DLC1 3'UTR with seed sequences highlighted in green at XbaI-SbfI sites (highlighted in red) in the pmirGlo reporter vector. (B) Sequencing analysis confirmed seed sequences cloned in the vector.

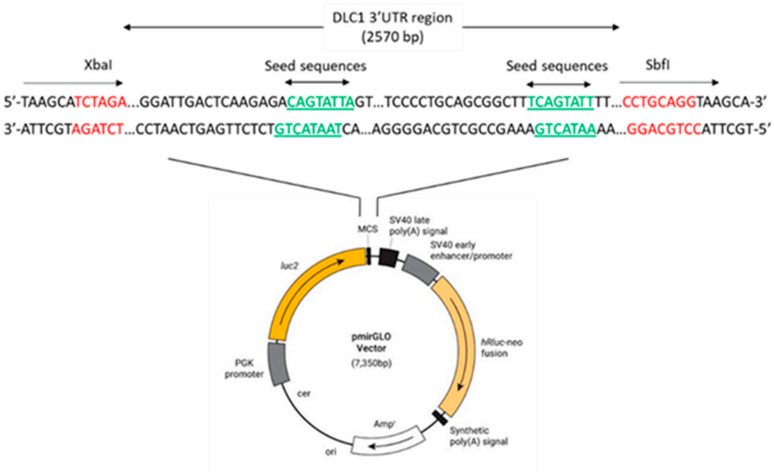

(**a**)

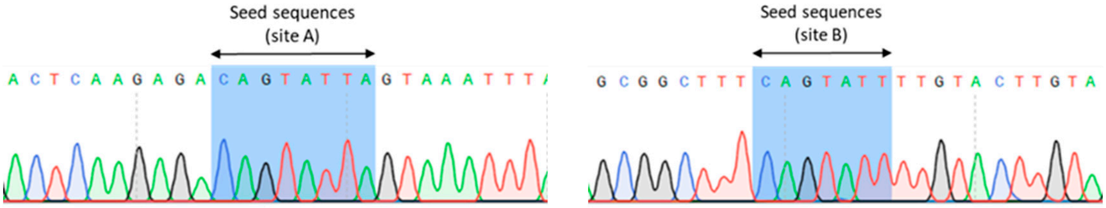

(**b**)

Supplementary Figure 4. (A) Representation of vectors with each of the binding sites constructed, namely WT-A and WT-B. (B) The constructs for luciferase activity of vectors containing, DLC1 3’UTR sequence, deletions of both site A (MT-A) and B (MT-B) or either site A or B (MT-AB). (C) Isolation of the binding site sequence (underlined) constructed into different vectors namely WT-A (46bp) and WT-B (53bp) together with mutant type vectors contained three substitutional mutation (red sequence) namely MT-A (46bp) and MT-B (53bp).

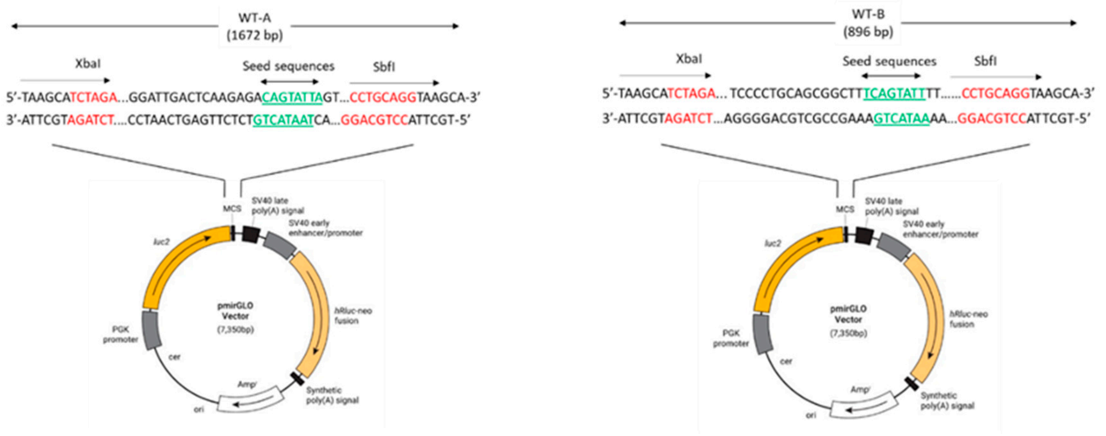

(a)

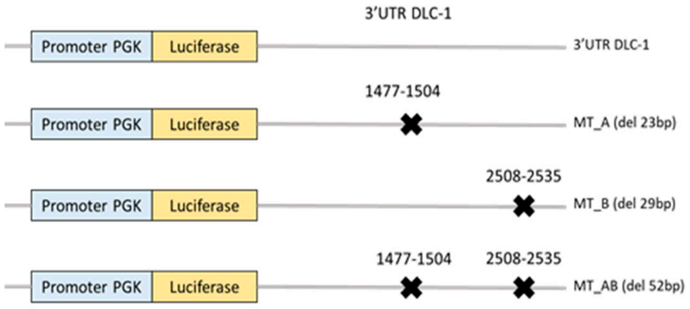

(b)

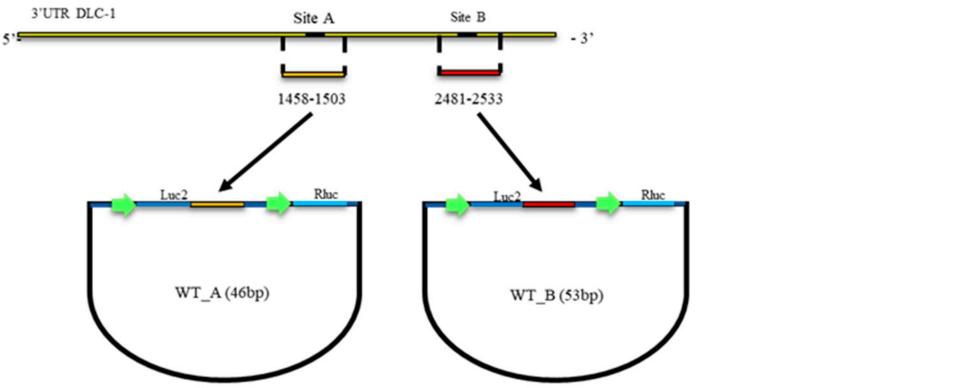

WT-A (46bp): 5’-…AAGAGACAGTATTAGTAAA…-3’ WT-B (53bp): 5’-…GCGGCTTTCAGTATTTTGTACT…-3’

MT-A (46bp): 5’-…ACTCAAGAGAGTCTATTAGTAA…-3’ MT-B (53bp): 5’-…CGGCTTTGTCTATTTTGTAC…-3’

(c)
